# The Cost of Relapsing-Remitting Multiple Sclerosis Patients Who Develop Neutralizing Antibodies during Interferon Beta Therapy

**DOI:** 10.1371/journal.pone.0159214

**Published:** 2016-07-08

**Authors:** Damiano Paolicelli, Sergio Iannazzo, Laura Santoni, Antonio Iaffaldano, Valentina Di Lecce, Alessia Manni, Vito Lavolpe, Carla Tortorella, Mariangela D'Onghia, Vita Direnzo, Elisa Puma, Maria Trojano

**Affiliations:** 1 Department of Basic Medical Sciences, Neurosciences and Sense Organs, University of Bari Aldo Moro, Bari, Italy; 2 SIHS Health Economics Consulting, Torino, Italia; 3 Biogen Italia, Milano, Italia; University of Oxford, UNITED KINGDOM

## Abstract

**Background:**

Relapsing Remitting Multiple Sclerosis (RRMS) patients treated with interferon beta (IFN beta) can develop neutralizing antibodies (NAbs) that reduce treatment efficacy. Several clinical studies explored the association of NAb+ status with increased disease activity.

**Objective:**

The aim of this study was to estimate the cost of RRMS patients who develop NAbs while treated with IFN beta by the Italian National Healthcare Service (NHS) and the Italian Society perspectives.

**Methods:**

The clinical data derived from a published observational study on 567 RRMS Italian patients treated with IFN beta. The management cost data derived from the published literature. Cost data were inflated to Euro 2014.

**Results:**

The annual direct cost to treat a patient was estimated in €15,428 in the NAb+ cohort and €14,317 in the NAb- cohort. The annual societal cost was estimated in €33,890 and €30,790 in NAb+ and NAb- patients, respectively. The cost increase related to the NAb+ status was €3,100 in the Italian societal perspective and €1,111 in the Italian NHS perspective.

**Conclusion:**

The results of this economic evaluation suggest the presence of an association between NAb+ status and increased costs for the management of RRMS in Italy. Further pharmacoeconomic research will be needed to confirm this first result.

## Introduction

The worldwide prevalence of Multiple Sclerosis (MS) is estimated at about 30 cases per 100,000 persons, with maximum values (over 100 cases per 100,000) in Northern Europe and North America. Data from the Italian Multiple Sclerosis Association (AISM) reported about 72,000 people with MS in Italy [1] The same data reported strong intra-regional variability, with the important case of Sardinia region, which presents an incidence rates among the highest in the world. MS is a disease with high social burden as it manifests mainly in the early adulthood and it has a chronic course. Consequently the disease maximizes both the social harm related to the gradual loss of productivity for individuals whose potential would normally be in the maximum phase, and health care costs related to the request of resources and assistance for the management of relapses and progressive long term disability. The social cost of MS in Italy is estimated in about 2.7 billion Euros per year [1] and the social cost per patient per year is estimated in about €38.000 [2].

The disease-modifying drugs (DMDs) for MS treatment include interferon beta (IFN beta) -1a and -1b. A problem that may occur during treatment with IFN beta is the development of neutralizing antibodies (NAbs), which block the interaction with IFN beta receptors [3], making the treatment largely ineffective. The incidence of NAbs was estimated in 2% to 45% in clinical trials of IFN beta [4]. The incidence of NAbs may vary depending on the specific product. Primary protein structure, absence of glycosylation, post-translational modifications and the presence of aggregates have been identified among the drug-related factors that affect immunogenicity [5]. To complicate the situation, it was noted that the state NAb positive (NAb+) is often transient.

The effect of the presence of NAbs on clinical outcomes has been investigated in several studies. A recent study investigated the effects of NAbs in a large cohort of RRMS patients treated with IFN beta in Bari University, Italy [6]. In this study the incidence of relapse was significantly higher in NAb+ vs NAb- cohort. The time to first relapse was significantly shorter in patients with NAb+ status. Moreover the study showed a trend towards a higher risk to reach the Expanded Disability Status Scale (EDSS) score 4, which represents a milestone for the disease progression [7].

The association of the presence of NAbs with a higher incidence of relapse and increased disease activity suggests an increased management cost of the MS. However, to our knowledge, no study in the literature investigated this aspect.

The objective of this analysis was to evaluate the management cost of RRMS patients who develop NAbs while on treatment with IFN beta, from the perspective of the Italian National Healthcare Service (NHS) and from the perspective of the broad Italian Society.

## Methods

The analysis was based on clinical data derived from the Italian observational study of the Bari University and on cost data derived from the published literature.

### Clinical data

The description of the observational study from which the clinical data derived is reported elsewhere [6]. In brief, a cohort of 567 RRMS patients who started treatment with IFN beta at the Centre for MS of the University of Bari (Italy) between 2005 and 2010 was analyzed. The presence of NAbs was evaluated throughout the follow-up. NAb+ patients were those with at least two consecutive positive samples (≥20 NU/ml) and were positive at their final assessment [8]. Patients who re-acquired NAb- status after two consecutive positive samples were defined reverting patients [9]. Patients who had at least one positive sample but failed to have this positivity confirmed in a consecutive sample evaluation were defined as fluctuating [8]. The following demographics and clinical data were recorded at the baseline: gender, age at the start of the IFN beta treatment, number of relapses in the previous 2 years, and disease severity evaluated with the EDSS score. During the follow-up the EDSS score at each visit was recorded, as well as the number of observed relapses. Patients received one of the different formulations of IFN beta at the standard dose: IFN beta-1b subcutaneously (SC) 250 mcg every other day in 14% of the cases, IFN beta-1a intramuscular (IM) 30 mcg once weekly in 38% of the cases, and IFN beta-1a 22 or 44 mcg SC three times weekly in 48% of the cases [6].

Reverting (n = 10) and fluctuating (n = 11) patients in the original dataset of the University of Bari study were not included in this economic evaluation.

Furthermore, to reduce casual differences in baseline characteristics between the NAb- and NAb+ cohorts a nearest-neighbor (NN) procedure was applied in STATA (STATA 13.1, Stata Corp College Station, Texas USA). This statistical procedure allowed the pairing between the NAb+ and NAb- patients in order to select two homogeneous subgroups [10,11]. The process was designed to perform an exact matching on gender and baseline EDSS covariates and the closest possible matching on age at IFN-beta assignment and number of relapses in the previous two years covariates. The comparison of the EDSS scores in time and of the number of relapses between the NAb+ and NAb- cohorts was performed on the NN matched population. The observation was restricted between 6 months from the baseline and the time point at which the fraction of patients lost to follow-up was above 15% (30 months after the baseline). The first 6 months were excluded to allow the complete onset of NAbs [5].

### Economic data

Cost data on the management of RRMS patients in Italy was derived from the literature. A targeted review of the published literature was performed to identify studies on cost of MS patient in Italy. Seven main studies were identified [2,12–17]. These studies presented a substantial heterogeneity in methods and results and it was thus impossible to proceed with a synthesis of the evidence. As a consequence a selection process was defined on the basis of the year of publication, completeness of the cost analysis (i.e. presence of direct and indirect costs) and number of patients enrolled. On the basis of these criteria, the study by Ponzio and colleagues was selected [2]. This study was designed as an observational cost of illness, using a societal prospective, with the information directly collected from a sample of patients through anonymous questionnaires. 1,686 patients were enrolled in Italian MS clinical centers (30%) or in rehabilitation units (10%) or among members of the Italian MS Association (AISM, 60%). The mean age of patients was 46.5 years. 47% of patients had RRMS. The majority of patients reported a mild (EDSS 0–3) and moderate severity (EDSS 4–6.5), respectively 40.8% and 44.5%. The remaining 14.7% reported a severe disability (EDSS ≥7). Mean total annual social cost per patient (Euro 2011) was €37,948, increasing for different severity: from €22,750 at an EDSS score of 0–3 to €63,047 at an EDSS score equal to or more than 7 [2].

The cost to manage one relapse was derived from the study from Kobelt et al., which was the only study that investigated explicitly this aspect in Italy [14]. The study provided an estimate of €4,000 for the social management of a relapse event. To the purpose of the economic analysis the total cost of relapse was split in direct and indirect cost. The direct cost of one relapse, supported by Italian NHS, was €2,339 and the direct/indirect cost, not supported by NHS, was €2,369 (both inflated to Euro 2014).

In general, the cost of MS can be divided into direct healthcare costs, direct non healthcare costs and indirect costs. The first category includes the healthcare costs directly related to the disease, such as the costs for hospitalization, outpatient visits and tests, rehabilitation and drugs. The category of direct non healthcare costs includes the costs directly related to the disease but that are not healthcare costs, such as transportation, home or car adaptation, special utensil or devices, walking aids and non- professional assistance (informal care). Indirect costs may relate to lost productivity or absence from work.

Therefore, for the purpose of the economic analysis and on the basis of the mentioned subdivision, the management costs reported in Ponzio study were grouped in:

Direct healthcare costs (except DMDs cost) plus direct non healthcare costs (only walking aids and wheelchair) that are sustained by the Italian NHS;

Direct non healthcare costs (except walking aids and wheelchair) plus indirect costs that are not sustained by the Italian NHS.

Values were inflated to Euro 2014 applying the consumer price index (Table 1) [18]. DMDs costs were removed from the total management costs, because the list of the products considered was not reported in Ponzio study and replaced by the weighted average cost of IFN beta products, calculated on the base of the ex-factory pack prices (including mandatory reductions and hidden negotiated discounts) [19–24] and the use ratio in the University of Bari study (Table 2) [6].

**Table 1:** Annual per patient costs to manage MS patients (DMDs excluded). Elaborated from Ponzio et al. [2]. Costs were inflated to Euro 2014 [1,8].

Annual per patient costs (Euro)	EDSS 0–3	EDSS 4–6.5	EDSS 7–9
Direct healthcare costs (except DMDs) + direct non healthcare costs (walking aids and wheelchair)	3,272	8,761	12,975
Indirect costs + direct non healthcare costs (except walking aids and wheelchair)	11,843	30,927	51,105

DMDs: disease-modifying drugs

EDSS: Expanded Disability Status Scale.

**Table 2:** Ex-factory prices of IFN beta and ratio of use.

Product	Ex-factory pack price* (€)	Ratio of use [6]	Source of ex-factory pack price
IFN beta 1b, 250 mcg qod	856.01 (15 vials, 250 mcg)	14.1%	Sup 250 GU 279, 2007 [19]; GU 127, 2000 [20]
IFN beta 1a, 30 mcg qw	790.17 (4 vials, 30 mcg)	37.9%	GU 11, 2004 [21]; GU 272, 2011 [22]
IFN beta 1a, 22 mcg tiw	764.36 (12 vials, 6 mln unit)	39.5%	Sup 154 GU 196, 2009 [23]; GU 274, 2011 [24]
IFN beta 1a, 44 mcg tiw	1,027.75 (12 vials, 12 mln unit)	8.5%	Sup 154 GU 196, 2009 [23]; GU 274, 2011 [24]

* excluding mandatory reductions and hidden negotiated discounts

mln = million; qod = every other day; qw = every week; tiw = 3 times a week.

## Results

In the cohort of 546 patients, 75 were NAb+ (13.7%) and 471 were NAb- (86.3%) (Table 3). The NN-matching process returned a cohort of 67 NAb+ and 59 NAb- patients. Eight patients in the NAb+ cohort could not be matched with any patient in the NAb- cohort. Eight patients in the NAb- cohort were matched with two patients in the NAb+ cohort. At baseline the mean (±SD) EDSS score was 2.45 (±0.73) in the NAb- cohort, and 2.40 (±0.71) in the NAb+ cohort. In the last 2 years before the beginning of IFN beta treatment the average number of relapses was 2.0 (±1.0) in the NAb- cohort, and 2.1 (±1.0) in the NAb+ cohort. The mean time of follow-up was 5.9 (±2.3) years for the NAb- cohort, and 5.2 (±2.5) years for the NAb+ cohort (Table 3).

**Table 3:** Baseline characteristics of the entire cohort and of the NN-matched cohort.

	Overall cohort		NN-matched cohort	
Baseline characteristics	NAb-	NAb+	NAb-	NAb+
N	471	75	59	67
Male sex, N (%)	146 (31%)	21 (28%)	17 (28.8%)	17 (25%)
Age at IFN beta enrolment; mean years (±SD)	31.7 (±8.7)	35.5 (±8.1)	34.9 (±7.3)	35.8 (±8.1)
EDSS score; mean (±SD)	2.3 (±0.9)	2.4 (±0.9)	2.4 (±0.7)	2.4 (±0.7)
EDSS score distribution (%)				
EDSS 0–1	9.6	5.4	0.0	0.0
EDSS 2	48.0	44.6	49.2	49.3
EDSS 3	25.5	29.7	32.2	32.8
EDSS 4	15.1	16.2	18.6	17.9
EDSS 5	1.3	4.1	0.0	0.0
EDSS 6	0.4	0.0	0.0	0.0
EDSS 6.5	0.0	0.0	0.0	0.0
EDSS 7	0.2	0.0	0.0	0.0
EDSS 8–9	0.0	0.0	0.0	0.0
Relapses in 2 previous years; mean N (±SD)	2.2 (±1.2)	2.0 (±1.0)	2.0 (±1.0)	2.1 (±1.0)
Follow-up; mean years (±SD)	6.2 (±2.5)	5.0 (±2.4)	5.9 (±2.3)	5.2 (±2.5)

NAb: neutralizing antibody

EDSS: Expanded Disability Status Scale.

**Fig 1:**
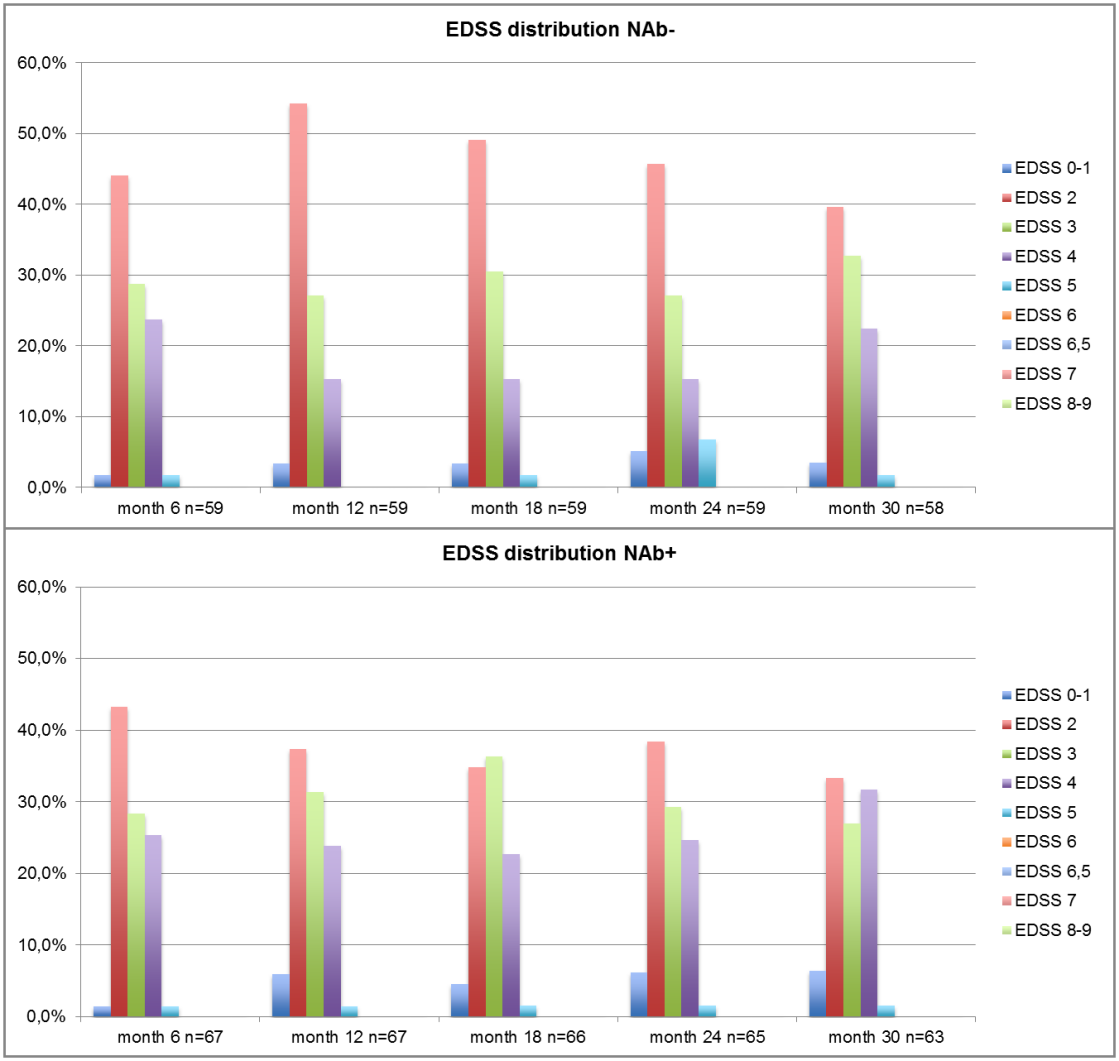
Distribution of the EDSS score in NAb- and NAb+ patients in the NN-matched cohort.

The comparison of the EDSS scores in time and of the number of relapses between the NAb+ and NAb- cohorts was performed on the NN matched population with a time horizon ranging from 6 months to 30 months after the baseline. After this date more than 15% of the NAb+ cohort was lost to follow-up (at month 36 only 56 patients over 67 were still on study) (Fig 1). To the purpose of the cost evaluation, the average time occupation of EDSS score strata during the observation (months 6–30) was estimated in the NAb+ and NAb- patients. In order to apply annual cost data from the study of Ponzio et al., EDSS score was stratified into three strata (EDSS 0–3, EDSS 4–6.5, EDSS 7–9) (Table 4). The mean (±SD) number of relapses per year observed during the follow-up was 0.28 (±0.32) in the NAb- patients, and 0.61 (±0.48) in the NAb+ patients.

**Table 4:** Time occupancy (i.e. percent of the total time in observation) of EDSS score strata defined according to the cost study of Ponzio et al. [2] in the NN-matched cohort.

	EDSS 0–3	EDSS 4–6.5	EDSS 7–9
**NAb-**	**79.3%**	**20.7%**	**0.0%**
**NAb+**	**72.9%**	**27.1%**	**0.0%**

NAb: neutralizing antibody

EDSS: Expanded Disability Status Scale.

In the NN-matched cohort, the annual direct cost to manage a cohort of NAb- RRMS patients resulted of €14,317. The same direct cost for the NAb+ patients was €15,428, with an increase of €1,111 per year specifically related to the development of NAbs. This increase was due to the increased number of relapses (68.4%) and to the increased need for healthcare resources (31.6%). In the perspective of the broad Italian society the total social cost increase related to the development of NAbs was €3,100 per patient-year Annual total costs were lower for the NAb- vs. NAb+ patients on all parameters. As a sensitivity analysis, the same cost analysis was repeated in the overall unmatched population. The differences in costs linked to the development of NAbs were less enhanced than in the NN-matched cohort, due likely to casual differences in baseline characteristics between the NAb- and NAb+ cohorts. (Table 5). However, also in the overall unmatched population it was confirmed the trend of NN-matched cohort with an increased cost related to the development of Nabs.

**Table 5:** Estimate of the annual total costs for the NAb- and NAb+ patients in the perspective of the Italian National Healthcare Service (direct costs) and the perspective of the Italian broad society (societal costs).

	Overall cohort (N = 546)			NN-matched cohort (N = 126)		
Cost Type	NAb-	NAb+	Increment	NAb-	NAb+	Increment
Total direct healthcare costs + direct non healthcare costs (walking aids and wheelchair)	€14,457	€15,457	€1,000	€14,317	€15,428	€1,111
Total indirect costs + direct non healthcare (except walking aids and wheelchair)	€16,959	€18,564	€1,605	€16,473	€18,462	€1,989
**Total social costs**	**€31,416**	**€34,022**	**€2,606**	**€30,790**	**€33,890**	**€3,100**

NAb: neutralizing antibody.

## Discussion

The objective of this study was to estimate the costs of the RRMS patients treated with IFN beta who develop NAbs. To this purpose the study was based on real world clinical data derived from an observational cohort of RRMS patients from a previously published observational study [6] and on literature costs data [12–17]. The observational cohort was studied in terms of disease progression and relapse rate to identify a relationship with the presence of NAbs. Then the average cost to manage RRMS patients in Italy as a function of disease severity (EDSS) and the cost to treat a relapse event were obtained from the review of the published literature. With this approach it was possible to estimate the annual cost of managing RRMS patients with and without NAbs. The results obtained showed that, as a consequence of the onset of NAbs the cost to manage RRMS patients can increase of €1,111 per patient-year from the Italian NHS prospective and of €3,100 per patient-year from the Italian societal perspective. This result may have implications in clinical decisions for physician and patients with RRMS, as the early adoption of a treatment less immunogenic can have the potential for better patient’s quality of life (QoL) and for cost savings for the NHS and the Society perspectives.

We have extrapolated these finding to the entire population of RRMS patients treated with IFN beta in Italy. In Italy a total of 72,000 MS patients is estimated [1] and about 65% of them have the RRMS form [25]. Of these 46,800 RRMS patients we estimated that 23,248 are treated IFN beta, based on the extrapolation of market sales data [26]. The incidence of NAbs was estimated with a large variability (from 2% to 45%) in clinical trials of IFN beta [4]. Assuming the proportion of NAb+ patients over the total in the University of Bari study (13.7%) [6] as a proxy for the incidence of NAbs in Italy, we estimated a total of 3,193 patients developing NAbs during IFN beta treatment in Italy. The annual cost increase specifically linked to their NAb+ status could thus be estimated in €9,897,374 from the Italian societal perspective of which €3,546,672 from the Italian NHS perspective.

This economic evaluation is funded on the clinical data of the University of Bari study [6] that confirmed the negative impact of NAbs on proper inflammatory clinical events such as relapse rate and time to a first relapse. It is key to consider that relapse is an exacerbation of the disease characterized by the appearance of new signs and/or neurological symptoms (or aggravation of existing ones) and related disabilities. A relapse has an important impact on the patient's daily life, his/her employment, social activity and QoL. Moreover a relapse is associated with considerable healthcare resources consumption as well as to high social costs determined by the productivity loss (working days lost and/or early retirement) and to increased direct non-healthcare costs (including informal care, house and car adaptation, etc.). The social cost of a relapse was estimated by Kobelt and colleagues in approximately €4,000 [14].

Furthermore, a relapse increases the level of EDSS (momentary or definitive), worsening the disability, which is linked to a reduction in the patient's QoL and increased costs.

The cost of illness study from Ponzio et al. showed that the severity of MS and poor QoL are strongly correlated with higher costs. The study reported that every additional EDSS point increases the total cost by 48% and that every improvement in EQ-5D scale determines a 15% decrease in total cost. The same study showed that the total annual mean costs per patient increased from €22,750 at an EDSS score of 0–3 to €63,047 at an EDSS≥7 [2].

To our knowledge, this economic evaluation is the first study attempting this assessment. However in 2014 a study was published that, with a different method, explored a similar concept [27]. The Walter study assessed the impact of NAb testing in RRMS patients treated with IFN beta in the Austrian setting. The analysis was based on a decision-analytic model and spanned over a time horizon of 5 years. The cost effectiveness of NAb testing versus no testing was evaluated. The results suggested that NAb testing reduces relapses and associated costs. The Walter study results seem in line with the outcomes of our study.

Same limitations can be ascribed to our economic evaluation, of whom the most relevant is undoubtedly the fact that the cost increase linked to the onset of NAbs was not directly observed in the same cohort of patients. The real world data of University of Bari study [6], in facts, due to the clinical nature of the used database was not suitable for this type of evaluation, and the detailed information on healthcare resources consumption that is necessary for proper costing was incomplete or missing. To this extent our study is to be considered a first attempt to explore the impact of NAbs from the economic point of view. A further consideration, related to the same limit, is linked to the analysis of treatments after the development of NAbs. The development of NAbs to IFN beta might influence treatment decisions in clinical setting and thus costs. More in detail, patients developing NAbs have to discontinue IFN beta therapy, as poor responders to that treatment, and switch generally to a more costly treatment. As this information is not available for the entire studied cohort it couldn’t be used in a costing perspective. In the light of this consideration, the results from this cost analysis are to be considered conservative, as they probably underestimate the cost impact of NAbs in RRMS patients. Another consideration is related to the NN algorithm, which did not match any patient at high (5+) or low (0–1) EDSS scores, limiting the range of applicability of the results to EDSS 2 to 4. However it is necessary to note that also in the overall unmatched population it was confirmed the trend of NN-matched cohort with an increased cost related to the development of Nabs. A further limitation may result from the fact that we were not able to capture the share of costs related to relapse of the Ponzio study. This could lead us to overestimate the total costs of relapse. However, this possibility should not have created a distortion in the analysis because, even if the costs of Ponzio study had included the cost of the relapse, the portion in excess would be the same in both NAb+ and NAb- patients costs.

As for most of the pharmaco-economic evaluations, further research is needed to overcome the limitations of the present study and confirm our conclusions.

The results of our economic evaluation confirm that the complication of the clinical picture, which occurs with the onset of NAbs during IFN beta therapy may have important economic implications, which can be crucial for policy decisions aimed at increasing the quality of life for patients as well as at promoting a more efficient use of economic resources.
